# Hunting for Early Melanomas With a Maximum Clinical Surface Diameter up to 6 mm: A Prospective Study of Reflectance Confocal Microscopy Features in 68 Consecutive Cases

**DOI:** 10.1111/cup.14731

**Published:** 2024-10-12

**Authors:** J. Pyne, S. MacDonald, S. Beale, E. Myint, S. Clark, A. Trang

**Affiliations:** ^1^ The University of New South Wales Sydney Australia

**Keywords:** dendritic cells, dermal papillary rings, epidermal disarray, melanoma, pagetoid cells and nests, reflectance confocal microscopy

## Abstract

**Background:**

Early diagnosis of melanoma and prompt effective therapy optimizes prognosis. Reflectance confocal microscopy (RCM) facilitates diagnosis by providing immediate 3D single cell resolution down into the papillary dermis.

**Methods:**

Consecutive cases were examined using a Vivascope 1500 confocal microscope at a single referral medical practice in Sydney, Australia 2019–2023. Melanoma clinical surface diameters were recorded by 0.1 mm increments up to 6.0 mm. The RCM features recorded were: pagetoid single cells or nests, pleomorphic cell shape, atypical dendritic cells, non‐edged papillae, variation in melanocyte size and confluent sheets of cells. All cases required diagnostic agreement by two dermatohistopathologists using hematoxylin and eosin staining followed by SOX 10 and/or PRAME stains if required.

**Results:**

Total cases were 68: 38 males (mean age 57) and 30 females (mean age 64). Melanoma in situ (*n* = 65) compared to invasive melanoma (*n* = 3), all males, invasion depth (0.4–0.5 mm). Most frequent RCM features found in 50% or more of cases within all diameter increments were: pagetoid single cells *n* = 64/68 (94%), pleomorphic cell shape *n* = 63/68 (93%), epidermal disarray *n* = 58/68 (85%), and atypical dendritic cells *n* = 45/68 (66%). Non‐edged dermal papillae were *n* = 42/68 (62%).

**Conclusion:**

Melanoma RCM features were found throughout the diameter ranges. Confocal examination may facilitate early melanoma recognition in these ranges.

## 1 Introduction

The earlier the diagnosis is made on primary cutaneous melanoma the more optimal the prognosis. Effective early disease detection requires the identification of features characteristic for melanoma. Feature poor small diameter early melanoma cases are well known to be frequently difficult to recognize based on their clinical and dermoscopy appearance. Little has been quantified about the characteristics of reflectance confocal microscopy (RCM) features within small melanomas with a maximum clinical diameter up to 6 mm.

Clinical and dermatoscopic examination visualizes the features of skin in a horizontal plane at limited shallow depth without single cell resolution. Conventional histopathology “breadloafing” examines tissue in two dimensional vertical sections with single cell and nucleus detail. In comparison, RCM has the capability of providing immediate live images with single cell resolution from the skin surface down into the superficial papillary dermis to depths of 150–200 μm [1] in three dimensions. This capability has the potential to improve melanoma diagnostic accuracy by providing additional information on small size early cases displaying minimal diagnostic clinical, dermatoscopic, and histopathologic melanoma associated features [2, 3, 4].

Recent commentary has questioned the value of diagnosing melanoma when the clinical surface diameter is less than 6 mm [5]. The main purpose of this study was to quantify the RCM features of melanoma cases with a clinical maximum horizontal diameter of 6 mm or less. A secondary purpose was to examine if these features vary as melanoma maximum surface diameter increased in increments from less than 2.0 mm up to 6.0 mm.

## 2 Method

Consecutive melanoma cases were prospectively collected from routine workflow in a referral medical practice in Sydney, Australia from 2019 until 2023 inclusive. Ethics Approval was provided by the University of Queensland, Australia. All patients entered the study after providing informed consent. There were no exclusions of cases based on age or anatomic site. Cases with a previous partial biopsy or known previous therapeutic intervention thus potentially having a confounding influence on the appearance of case features were excluded.

Selection of each case for RCM examination was based on both clinical and dermoscopy assessment. Clinical assessment included the characteristics of the case plus comparison to other lesions based on the “ugly duckling” sign involving colors, shape and pattern or other less common features such as a halo. Dermoscopy was performed with both polarized and non‐polarized light using a Heine Delta 20T dermatoscope.

Prior to excision RCM examination of each case was conducted using a Vivascope 1500 microscope. The confocal features recorded are set out in Table 3. Rather than using a vertical stack of images with preset separation the entire tumor mass was examined by manual adjustment of the horizontal inspection plane. Each case was examined using a manufacturer supplied single use plastic cap stuck onto the skin over the relevant tissue. The dermoscopy image in the Vivascope 1500 microscope software provided a template to systematically examine the whole tumor mass in three dimensions.

Full excisional biopsy was the intention of every case. Cases were selected for excision based on the collective clinical, dermoscopy, and confocal features of each case. Prior to excision dermoscopy was used to measure each case maximum clinical tumor horizontal diameter in vivo by 0.1 mm increments using the Vivacam dermatoscope. At biopsy all cases were excised down to fat with a 2 mm dermoscopy identified peripheral clear margin using a Heine Delta 20T dermatoscope. The authors concede it is not certain all cases were fully excised in toto as some residual melanoma tissue may not have been able to be identified using dermoscopy just prior to the excision. However, we consider the volume of such residual tissue too small and infrequent to adversely affect the results. Minimum microscopic margins for the excisional biopsies in all cases were recorded to the nearest 0.1 mm.

Inclusion of each case required a histopathological diagnosis of melanoma with a maximum horizontal clinical diameter of 6 mm or less. All histopathologic diagnoses other than melanoma and melanomas with a maximum horizontal diagnosis of 6.1 mm or more were excluded. The excised tissue was prepared for histopathologic examination by “breadloafing” sections. Vertical transverse sections of tissue were selected at 2–3 mm intervals over the full length of the typical fusiform specimen for microscopic assessment. Excised tissue was then examined following hematoxylin and eosin staining as well as SOX 10 and PRAME stains if needed. Each case required two dermatopathologists, each blinded to the other, to agree on providing the diagnosis of melanoma. A dermoscopy image of each case was provided to each dermatohistopathologist. RCM images were also made available to the reporting dermatohistopathologists on request. If only one of the two dermatohistopathologists diagnosed melanoma these cases were also excluded.

## 3 Limitations

At the time of excisional biopsy nearly all cases displayed prominent brown during clinical examination. Only one case has mostly pink with minimum brown. There were no cases displaying pink as the only color present being selected for excisional biopsy. Melanomas with minimal clinical evidence of melanin may have been missed or at least underrepresented in the cases being selected for excisional biopsy. Cases characterized by displaying a darker brown color seen on clinical or dermatoscopic examination may have been more “eye catching” and may be overrepresented.

Melanomas in this study were predominately on fair skin Australian born patients typically displaying pronounced chronic solar damaged skin. Melanomas with lentiginous histopathology may be overrepresented. Patients with Fitzpatick 1 and 2 skin types numbered 61, seven patients had Fitzpatrick 3 skin. There were no patients with Fitzpatrick 4 or above skin in this study.

The authors acknowledge that Langerhans cells and melanocytes with multiple dendritic processes can have overlapping morphologic appearances. We assessed the following morphological features to favor dendritic cells being malignant melanocytes: enlarged cell size (particularly over 20 μm diameter), prominent variable irregular cell shape, thickened dendritic processes, a variable diameter along a given dendritic process, elongated dendritic length, and atypia of the nucleus (increase in nuclear to cytoplasm volume ratio, atypical nucleus shape and substantial variation in the nuclear size). Additional immunoperoxidase stains could be used to clarify which dendritic cells are either melanocytic or Langerhans. However, we considered this investigation more applicable to further future study rather than within the scope of this study.

## 4 Results

**FIGURE 1:**
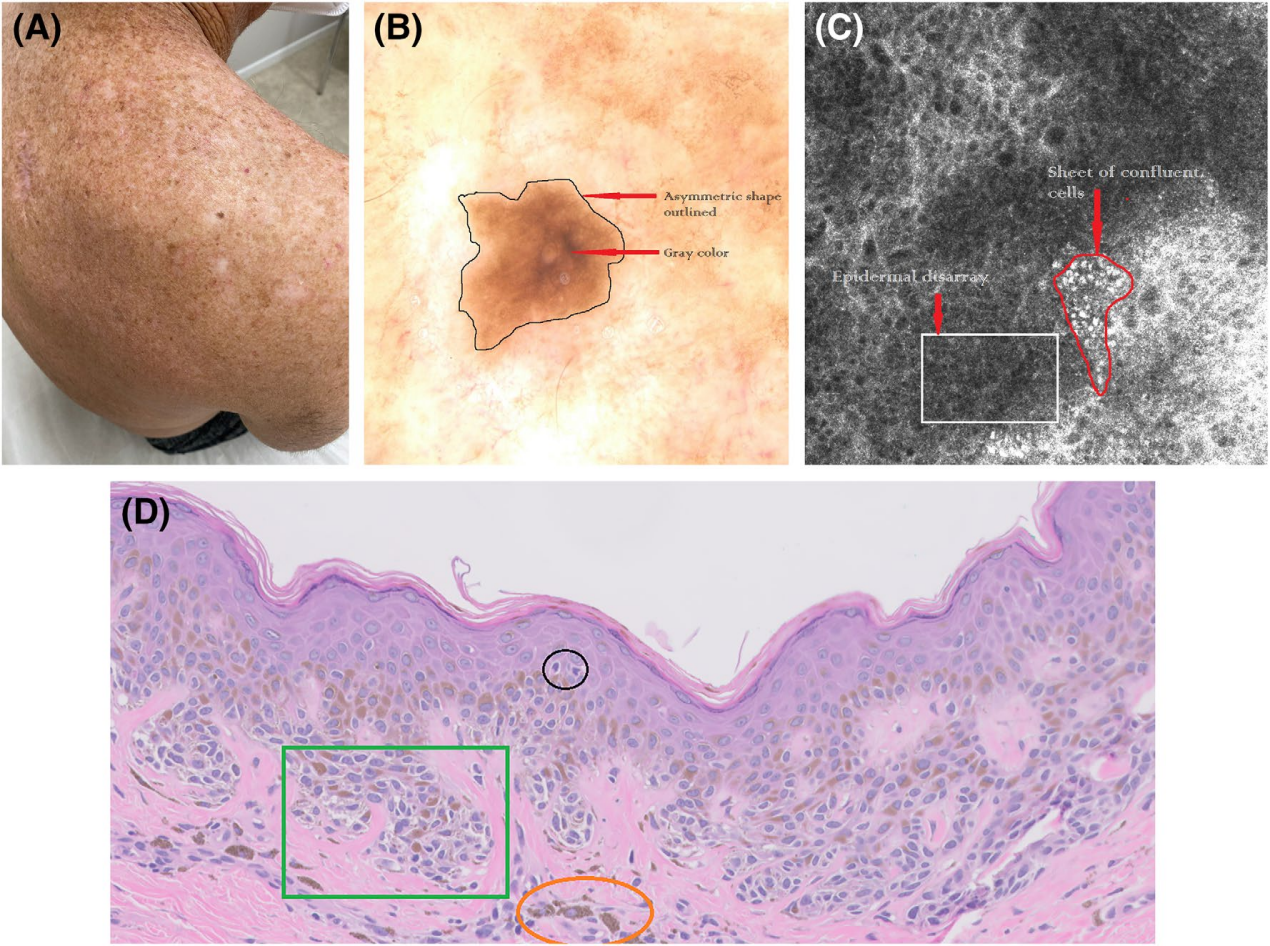
(A) Clinical image of a melanoma in situ on the right upper back of a 59 year old male, (B) Vivacam dermatoscopic image [10× magnification], the same melanoma in situ presenting as a brown macule is outlined by a black line and displaying central gray color, (C) Vivascope RCM image featuring epidermal disarray within a white rectangle and a confluent sheet of cells outlined by a red line [200× magnification] and (D) hematoxylin and eosin stain: within the black circle are two single pagetoid melanocytes, in the green rectangle are nested pleomorphic melanocytes and within the orange oval macrophages containing brown melanin pigment in the papillary dermis [200× magnification].

A total of 68 cases (where both dermatohistopathologists declared a melanoma diagnosis) were collected, 38 on males (mean age 57) and 30 on females (mean age 64), see Table 1. All cases presented as macules with no overt surface elevation. Seven other separate cases were excluded from the study where only one of the two independent pathologists diagnosed melanoma. These seven cases were typically in younger patients displaying combinations of clinical, dermoscopy, and RCM features shared both nevi and melanoma. It was not possible to separate which of these seven cases were either atypical nevi or feature poor melanoma so they were excluded from the study. The clinical, dermatoscopic, confocal, and histopathologic features of a typical MIS case with a clinical surface diameter of 4.8 mm is set out in Figure 1.

**TABLE 1:** Patient characteristics by age, sex, and clinical or in vivo surface diameter.

		Male (*n* = 38)	Female (*n* = 30)
Age (years)	Range (min, max)	18–78	36–90
	Mean	57	64
	IQR	47–67	51–75
Melanoma in vivo surface diameter (mm)	< 2.0	4	0
	2.0–3.0	9	8
	3.1–4.0	5	9
	4.1–5.0	11	6
	5.1–6.0	9	7

Table 2 sets out the study cases by anatomic site and sex. Males recorded most cases on the back (*n* = 13) followed by chest (*n* = 6). On females the respective most frequent sites were back (*n* = 10) then knee or leg (*n* = 8). No cases were recorded on the following sites: scalp, eyelid, cheek, chin, cutaneous or mucosal lip, hand, genitals, fingers, and toes. No patients were known to be immunosuppressed.

**TABLE 2:** Melanoma cases by anatomic site and sex.

Anatomic site	Male	Female
Ear	1	1
Forehead	1	0
Nose	1	0
Neck	1	1
Chest	6	3
Back	13 (1 invasive, 0.5 mm)	10
Upper arm	2	4
Forearm	3	0
Abdomen	2 (1 invasive, 0.5 mm)	1
Thigh	2 (1 invasive, 0.4 mm)	1
Knee and leg	5	8
Foot	1	1

*Note*: All cases were melanoma in situ except the three invasive cases as marked with Breslow invasion depth (mm).

Melanoma in situ was reported in 65 cases. The other remaining three cases were invasive and all on males: one with a Breslow of 0.4 mm (thigh) the other two cases (abdomen and back) both had a Breslow of 0.5 mm. For males the minimum surface diameter recorded for a melanoma in situ on males was 2.3 mm and for the invasive cases 4.4 mm. The minimum surface diameter for females was 1.6 mm. All three invasive cases on males had no tumor induced ulceration. One invasive case on the abdomen had a mitotic count of 1 per mm^2^ the other two cases (thigh and back) had a zero mitotic count. This study did not record any cases of acral, nodular or desmoplastic melanoma. Of the total 68 cases, eight (*n* = 8/68, 12%) were reported with involved lateral margin(s). These cases typically displayed lentiginous spread of melanocytes along the dermoepidermal junction.

**FIGURE 2:**
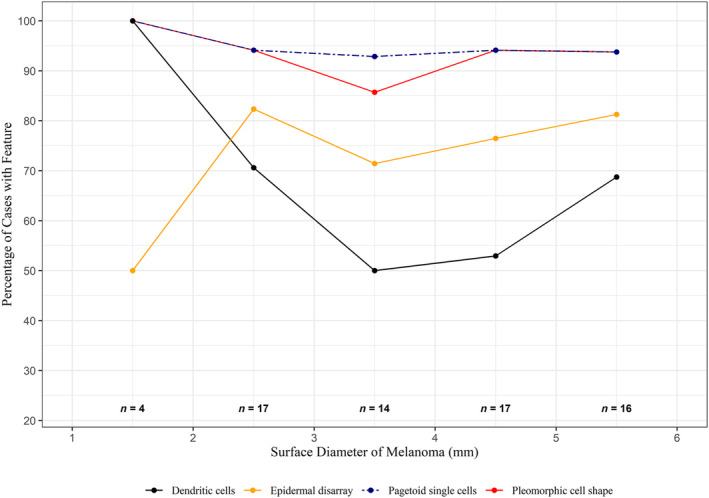
RCM features with high prevalence in melanoma cases.

**FIGURE 3:**
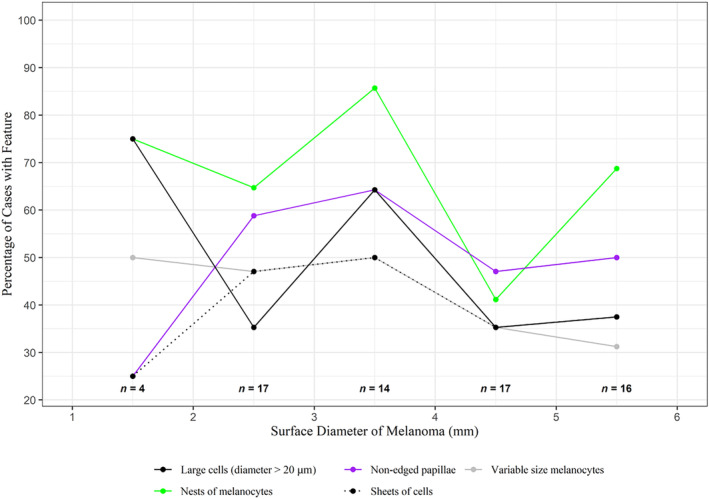
RCM features with moderate prevalence in melanoma cases.

**FIGURE 4:**
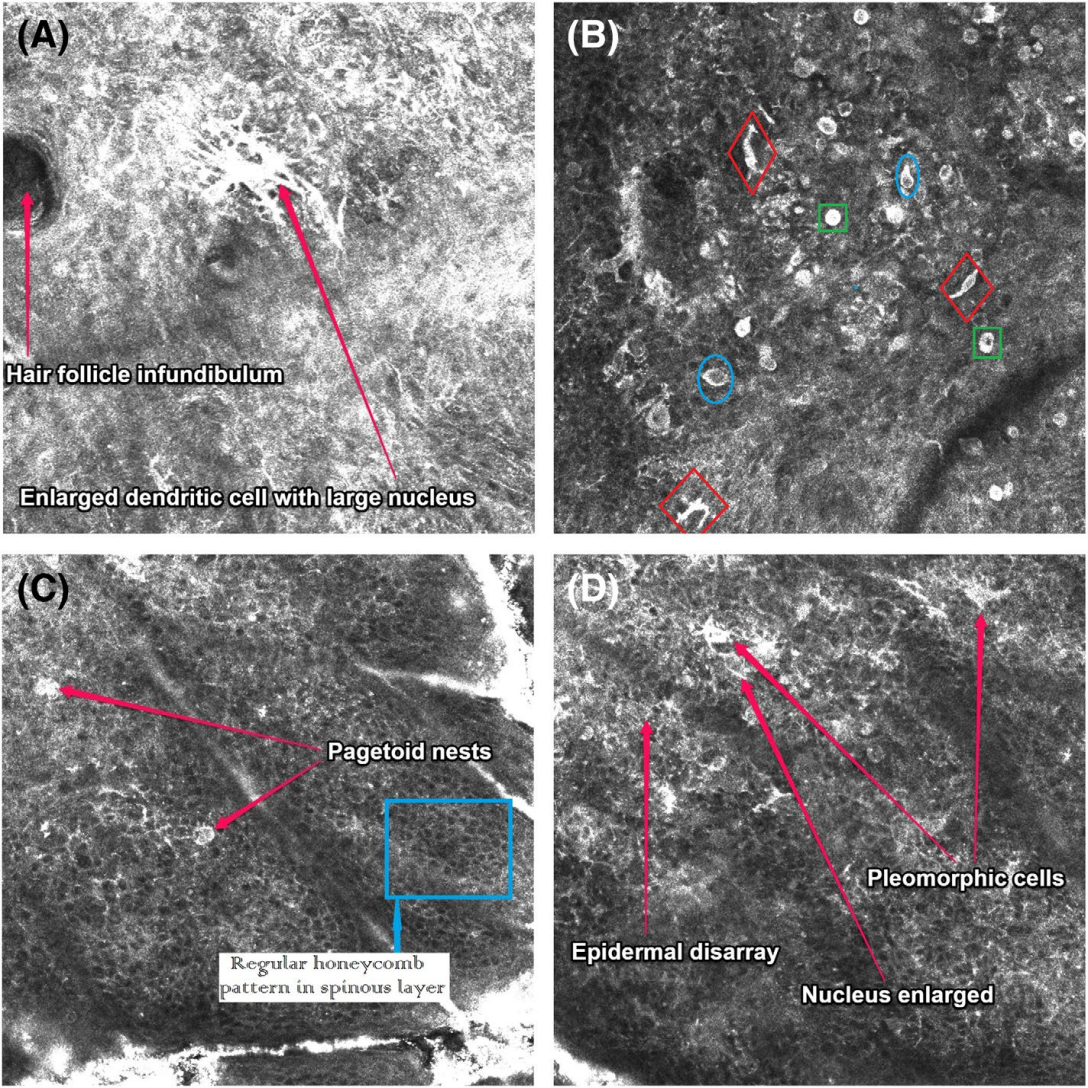
RCM images: all images 200× magnification, (A) illustrating a hair follicule infundibulum and adjacent enlarged atypical dendritic cell at level of the dermoepidermal junction (DEJ), (B) at the base of the spinous layer in the epidermis: atypical dendritic cells (within red diamonds), pagetoid single cells (within green squares) and atypical cells with an increased nuclear to cytoplasm ratio (inside blue ovals), (C) in the mid spinous layer: pagetoid nests and in a separate area background regular honeycomb pattern created by keratinocytes (within blue rectangle), and (D) the transition from the DEJ up into the spinous layer: epidermal disarray, pleomorphic cells, and nuclear enlargement.

**FIGURE 5:**
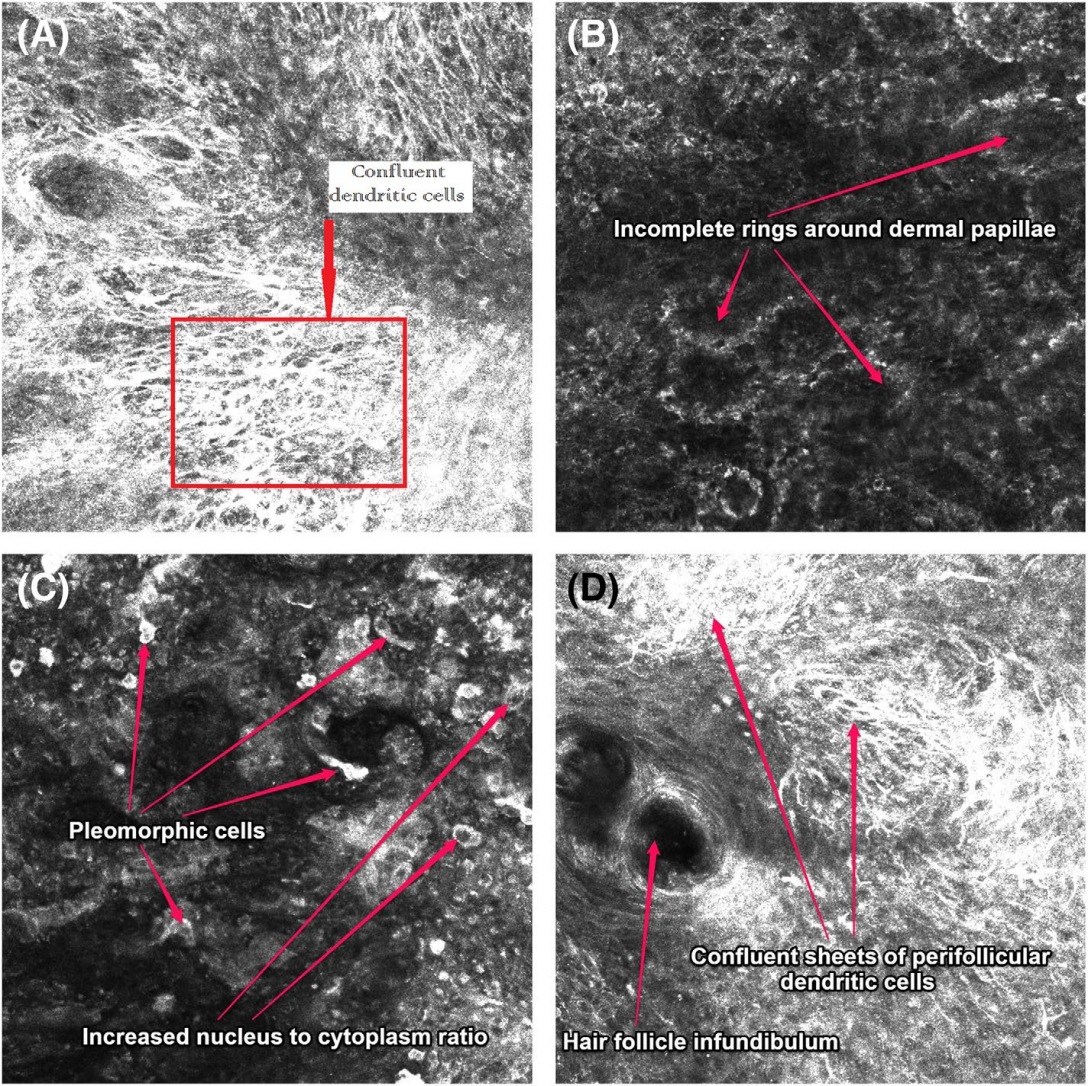
RCM images: all images [200× magnification], (B) at the level transecting some of the dermal papillae, (A), (C) and (D) all at the dermoepidermal junction, (A) displays confluent atypical dendritic cells, (B) incomplete rings around dermal papillae, (C) atypical pleomorphic cells and cells with increased nucleus to cytoplasm ratios, and (D) hair follicule infundibulum with adjacent confluent sheets of perifollicular dendritic cells.

Figures 2 and 3 set out the RCM features found to have respective high and moderate prevalence. These same RCM features identified in the study cases are illustrated in Figures 4 and 5. Males and females both recorded the most frequent confocal features in the same descending order: (1) single pagetoid cells, (2) pleomorphic cell shape, (3) cells with dendritic morphology, and (4) epidermal disarray, see Table 3. When male and female data were combined this order of frequency was maintained across the diameter range from 2 up to 6 mm, see Figure 2. There were also no significant differences in the lower frequency features when comparing males and females, again see Table 3.

**TABLE 3:** Confocal features range of prevalence by sex.

Confocal features (percentage of cases per feature)	Overall frequency (%) *n* = 68	Males *n* = 38	Females *n* = 30
Pagetoid single cells	93–100	89–100	86–100
Pleomorphic shaped cells	86–100	91–100	78–100
Dendritic cells	50–100	55–100	44–75
Epidermal disarray	50–82	50–89	75–100
Nests of melanocytes	41–86	45–80	33–89
Non‐edged papillae	25–64	25–80	50–63
Variation in melanocyte size (10–20 μm)	31–50	22–50	43–56
Large atypical cells (> 20 μm)	35–75	33–75	25–78
Sheets of cells	25–50	25–60	33–44
Pagetoid nests of cells	0–25	0–25	0–22
Large round cells	6–25	0–25	0–17

**FIGURE 6:**
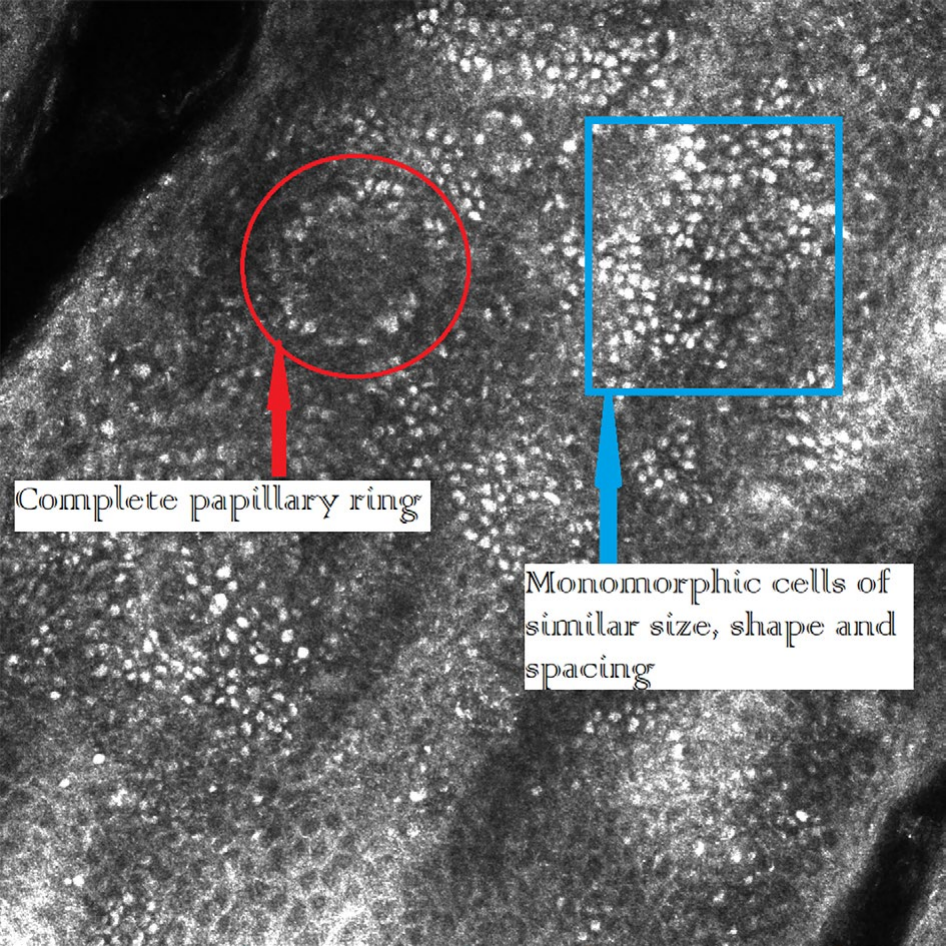
RCM images of a junctional nevus [200× magnification]. The red circle surrounds a complete papillary ring and within the blue square are the monomorphic cells of similar size, shape, and spacing.

In contrast to the RCM melanoma features displayed in Figures 1, 4, and 5, the typical characteristic RCM appearance of a junctional nevus are seen in Figure 6. Table 4 sets out the minimum microscopic margins for all cases.

**TABLE 4:** Minimum microscopic margins for the excisional biopsies.

Microscopic margins (mm)	Number of cases (total, *n* = 68)
Margin involved	12 (17%)
0.1 to 0.5	10 (15%)
> 0.5 to 1.0	12 (17%)
> 1.0 to 1.5	15 (22%)
> 1.5	19 (28%)

## 5 Discussion

Dermoscopy and confocal images were deliberately provided to the reporting dermatohistopathologists with the intention to maximize diagnosing small size feature poor cases. This methodology [6] may increase the detection of small dimension feature poor cases resulting in a high proportion of MIS cases compared to invasive disease. We recorded high numbers of MIS (*n* = 65) relative to invasive melanoma (*n* = 3) in our study cohort (*n* = 68) compared to other studies [3, 7, 8]. The five most frequent RCM features within melanoma had a similar order and frequency to previous studies [3, 7, 8] as set out in Table 5.

**TABLE 5:** Comparison with other similar RCM studies.

RCM feature study	Pagetoid cells	Atypical cells	Epidermal disarray	Dendritic cells	Nests of melanocytes
Pellacani et al. [5] Diameters not listed MIS *n* = 42 MM *n* = 94	*n* = 106/136 77.9% Roundish cells only recorded	*n* = 74/136 54.4% Pagetoid cells only recorded	*n* = 85/136 62.5% Superficial epidermis only	*n* = 75/136 55.1% Pagetoid cells only	*n* = 72/136 52.9% Only recorded in dermis
Guitera et al. [7] Diameters not listed MIS *n* = 133 MM *n* = 103	*n* = 80/113 70.8%^a^ Only MIS shown here	*n* = 91/113 80.5% Only MIS shown here	*n* = 71/113 62.8% Only MIS shown here	*n* = 57/113 (50.4%) Only MIS shown here	*n* = 34/113 30.1%^b^ Only MIS shown here
Pupelli et al. [3] Diameters 5 mm or less MIS *n* = 11 MM *n* = 13	*n* = 11/24 (46%) MIS + MM combined^c^	*n* = 20/24 (83%) MIS + MM combined	*n* = 11/24 (46%) MIS + MM combined	*n* = 5/24 (38%) MIS + MM combined	*n* = 19/24 (79%) MIS + MM combined
This study Diameters 6 mm or less MIS *n* = 65 MM *n* = 3	*n* = 64/68 (94.1%) MIS + MM combined	*n* = 63/68 (92.6%) MIS + MM combined	*n* = 52/68 (76.5%) MIS + MM combined	*n* = 44/68 (64.7%) MIS + MM combined	*n* = 44/68 (64.7%) MIS + MM combined

^a^ Only dendritic pagetoid cells listed (Guitera et al. [7]).

^b^ Only Junctional nests listed.

^c^ Melanoma in situ (MIS) plus invasive melanoma (MM) cases combined total shown.

The higher frequency of features recorded in our study may be due to a more thorough examination of the whole tumor mass by RCM manual scanning rather than using a preset vertical stack of images displaying only parts of the total tumor mass. Dermoscopy alone without RCM has been recently described as suboptimal in diagnostic accuracy when detecting melanoma with diameters 5 mm or less [9].

The consistent frequencies of RCM features across the diameters from 2 to 6 mm inclusive in Figures 2 and 3 suggest melanomas in this range of diameters may have similar ease in diagnostic recognition in similar cohorts. To the best of our knowledge these RCM feature findings have not been previously quantified in such small diameter range early disease cases. We examined all tissue down to 200 μm underneath the dermoscopy identified margin of each case. In comparison, when using vertical stack imaging not all tissue is visualized. Only four cases were recorded with diameters of 2 mm diameter or less. The frequency of the recorded RCM features observed in these tiny cases may not be representative of other cases of this size not in our study.

In our cohort the back stood out as the most frequent site for melanoma on both males and females. This is consistent with other studies [10] and not surprising given the relatively large surface area on the back compared to other sites.

A frequent anecdotal RCM observation in this study was an increased nucleus size within atypical and enlarged cells within the melanoma tumor mass and an increase in the ratio of the size of the nucleus compared to the size of the same cell cytoplasm, see examples in Figure 5C. Nuclei size and the ratio of nucleus to cytoplasm size could be further quantified by future investigation. Further, the nucleus in melanocytes of nevi typically display a round or oval shape and typically have a central intracellular location. In contrast, we noticed the nucleus within malignant melanocytes is often an asymmetrical shape and located in a more eccentric intracellular location. Another anecdotal observation in our cohort was that malignant dendritic melanocytes were noted to have elongated dendritic processes with more variable morphology dimeters compared to the dendritic cells seen in nevi. The extent to which dendritic cells in melanoma or nevi are either Langerhans cells or melanocytes has not been quantified.

The horizontal diameter of atypical cells in the epidermis and superficial papillary dermis can be accurately measured in a single horizontal focal plane by RCM. However, the vertical diameter measurement has considerable variation in depth and cannot be measured in the horizontal plane. We did not record horizontal atypical cell diameters in our study. Pagetoid cell diameters greater than 20 μm and been recorded with higher frequency in melanoma: *n* = 110/135, 80.9% compared to nevi *n* = 51/215, 23.7% OR 11.2 (95% CI 6.64–18.9) [5].

Epidermal disarray estimation is subjective and there is a spectrum of disorder from mild to severe in this disarray. We observed this disarray to be more frequent and further developed in the basal layer and lower spinous layer. The precise spatial distribution of the disarray was not formally recorded. The grade of disarray could also be quantified with future work. We recorded the presence of RCM features only. Future investigation on the spatial arrangement vertical stack. The distribution of RCM features within small dimension melanomas may also reveal additional information with diagnostic relevance via clinicopathologic correlation.

Atypical dendritic cells, non‐edged papillae, epidermal disarray and cell shape pleomorphism were all recorded with frequencies above 50% across all clinical diameters up to 6 mm, see Figure 2. These findings suggest RCM including the exchange of images may assist in clinicopathological correlation during the diagnostic process.

The use of dermoscopy (no cellular resolution seen) and RCM in this study was to facilitate diagnostic excision rather than margin assessment for therapeutic excision. Given dermoscopy and RCM are both three dimensional examination compared to the two dimensional vertical sections of routine histopathologic examination it is not surprising the minimum microscopic margins results did not show consistent clear margin measurements.

## 6 Conclusion

Within melanomas in our cohort we found the following RCM features: single pagetoid cells, pleomorphic cell shape, atypical dendritic cell morphology and epidermal disarray to all be present at frequencies over 50% across the full range of surface diameters up to and including 6 mm. These features appear to be RCM hallmarks for small melanoma with diameters ranging up to 6 mm.

## Conflicts of Interest

The authors declare no conflicts of interest.

## Data Availability

The data that support the findings of this study are available from the corresponding author upon reasonable request.
